# Ecological histories govern social exploitation by microorganisms

**DOI:** 10.1093/ismejo/wrae255

**Published:** 2024-12-23

**Authors:** Kaitlin A Schaal, Pauline Manhes, Gregory J Velicer

**Affiliations:** Institute for Integrative Biology/Department of Environmental Systems Science, ETH Zurich, 8092 Zurich, Switzerland; Department of Evolution, Ecology and Behaviour, University of Liverpool, Liverpool, L69 7BE, United Kingdom; Department of Biology, Indiana University, Bloomington, IN 47405, United States; Pavillon de Neurologie, CS 10217, CHU Grenoble Alpes, 38043 Grenoble, Cedex 14, France; Institute for Integrative Biology/Department of Environmental Systems Science, ETH Zurich, 8092 Zurich, Switzerland; Department of Biology, Indiana University, Bloomington, IN 47405, United States

**Keywords:** cooperation, cheating, ecological history, exploitation, resource level

## Abstract

Exploitation is a common feature of social interactions, which can be modified by ecological context. Here, we investigate effects of ecological history on exploitation phenotypes in bacteria. In experiments with the bacterium *Myxococcus xanthus*, prior resource levels of different genotypes interacting during cooperative multicellular development were found to regulate social fitness, including whether cheating occurs. Responses of developmental spore production to manipulation of resource-level histories differed between interacting cooperators and cheaters, and relative-fitness advantages gained by cheating after high-resource growth were generally reduced or absent if one or both parties experienced low-resource growth. Low-resource growth also eliminated exploitation in some pairwise mixes of cooperative natural isolates that occurs when both strains have grown under resource abundance. Our results contrast with previous experiments in which cooperator fitness correlated positively with concurrent resource level and suggest that resource-level variation may be important in regulating whether exploitation of cooperators occurs in a natural context.

## Introduction

Ecological context often modifies biological interactions, including those involving social exploitation, here conceived as gaining in absolute fitness from a social interaction (e.g. [1, 2]). One type of interaction involving exploitation is cheating, in which one individual contributes less to a cooperative phenotype than an interacting partner while benefitting disproportionately from that partner’s contribution (e.g. [3–5], see also [6]). The presence of cheats in a population may threaten its long-term stability and the fate of its cooperative traits if the cheats are obligate defectors, i.e. lacking functional cooperation genes [7, 8]. The degree to which such cheaters lower cooperation in a population may depend on the ecological conditions—past or present—experienced by the interactants. Indeed, abiotic ecology might determine whether cheating even occurs between given partners. Here, we investigate how two social interaction scenarios involving exploitation by bacteria—including cheating by obligate defectors—are impacted by past ecological conditions experienced by the interacting partners.

Our first exploitation scenario is cheating by obligate defectors, in which individuals unable to express a cooperative trait (or that can express it only at a low level) exploit those expressing it at a high level to gain a relative-fitness advantage over them [3, 5, 9]. Following Fig. 2A of [2], such individuals show a higher relative fitness than the cooperator during interactions, regardless of the absolute performance of the mixed population. Cheating by obligate defectors can reduce equilibrium levels of the cooperative trait in the overall population and might drive the trait to extinction [7, 10–14]. Severely defective cheaters might require exploitation of cooperators to avoid extinction [2, 7].

Our second scenario is exploitation between bacterial genotypes that are all capable of high levels of cooperation, at least in clonal groups. In this scenario, one cooperator genotype gains in absolute fitness by interacting with another, regardless of the absolute performance of the latter or the absolute effect on the mixed population (Fig. 2C of [2]). Competition between cooperation-proficient genotypes [1, 4, 15, 16]—including competition involving social exploitation as conceived here [1, 15]—can alter their relative frequencies in a population without lowering the overall frequency of genotypes capable of cooperation, and thus does not fundamentally threaten the persistence of cooperation.

Microorganisms exhibit diverse forms of cooperation [3–5, 17–31], but ecological context can influence the relative fitness of cooperators and those they interact with, such as cheaters [32–35]. For example, environmental toxins such as antibiotics [36] or heavy metals [37] can benefit cheaters, whereas oxidative stress [38] and viscosity [39] can benefit cooperators. High diversity [40], competitors [34], or predators [41] within communities can also benefit cooperators. In some cases, phage presence benefits cooperators [42, 43], but in others it benefits cheaters [44, 45].

Both nutrient identity [46] and level [32] during cooperation can impact cooperation’s benefits and costs [47]. For example, experiments with bacteria have tested for nutrient-level effects on cooperator and cheater fitness during cooperation mediated by diffusible public-good molecules. High resource availability during cooperation was found to increase cooperator fitness—apparently by decreasing the relative cost of cooperation compared to low-resource conditions—although cheating was not eliminated [32].

The relative fitness of cooperators may also be impacted by prior ecological conditions through lasting effects. Ecological histories are known to influence a variety of inter-specific interactions [48–51], suggesting that they may also influence the social fitness of microorganisms. The plausibility of this hypothesis is illustrated by experiments with the social amoeba *Dictyostelium discoideum,* which develops into spore-bearing fruiting bodies in response to starvation. *D. discoideum* cells can differ in their propensity to form spores during development due to variations in their nutritional histories [52–56]. For example, during starvation, *D. discoideum* previously grown in glucose media forms proportionally more spores than cells grown without glucose [54, 57, 58]. If co-developing defectors and cooperators were to differ in their nutritional histories, this would therefore impact their relative fitness as cells with a history of available glucose increase in frequency. Nutritional histories of co-developing cooperator and defector strains might also impact their relative fitness during development if genetic differences between strains are relevant to effects of nutrient-history variation on developmental phenotypes.

We investigate the effect of nutritional history on social exploitation in the soil bacterium *Myxococcus xanthus*, which forms spore-filled fruiting bodies upon starvation through a developmental process involving the exchange of several intercellular signals [59, 60]. Groups of tens-of-thousands of *M. xanthus* cells aggregate, but only a small minority become spherical spores; the remaining cells either die or remain rod-shaped cells [31, 61, 62]. In this system, developmental cheaters have been identified that are obligately defective at spore production, producing low yields in monoculture compared to high-sporulating cooperators, but which produce proportionally more spores than cooperators in mixed groups, thereby demonstrating direct social exploitation [3, 63]. Laboratory experiments have repeatedly shown that developmental cheaters can rapidly increase in frequency due to their exploitation of cooperators, in some cases leading to large population crashes and even extinction events [3, 7, 22]. Exploitation during development has also previously been observed between *M. xanthus* natural isolates that all sporulate at similarly high levels in monoculture [1, 64].

To further our understanding of the role of exploitative interactions in microbial evolution, we tested the effects of nutritional history on both cheating by obligate defectors and exploitation by cooperation-proficient genotypes. For the cheating scenario, we cultured a high-sporulating cooperator strain and two sporulation-defective cheater strains of *M. xanthus* in both high- and low-resource growth media before mixing the cooperator with each cheater genotype pairwise in all possible nutrient-history combinations and then quantified spore production by each genotype after starvation-induced development. We tested (i) whether nutrient-history variation impacts cooperator-cheater interactions, including whether cheating occurs or not, and (ii) whether any such nutrient-history effects are similar or different between the cheater-cooperator genotype pairs. This allowed us to account for genotype-specific responses to nutrient level. Similarly, for the scenario of exploitation between cooperation-proficient genotypes, we examined the effects of nutritional history on pairwise mixes of natural isolates in which one strain has been shown to exploit the other during development [1]. In contrast with the strains used for the cooperator-cheater interactions, these natural isolates have short histories of lab cultivation, and all sporulate at a high level in monoculture. They are genetically diverged from each other, allowing for the possibility of differential effects of nutritional history on their interactions.

## Materials and methods

### Terms

In this study, our adopted definitions of “exploitation”, “defection”, and “cheating” and their variants (see *Introduction*) are applied to overall patterns of quantitative phenotypes that have been observed in *M. xanthus*. In *M. xanthus*, all cheaters identified so far are obligate defectors who exploit a cooperative partner sufficiently to gain a relative advantage over that cooperator. (Cheating by behaviorally facultative defection, i.e. by reducing investment in cooperation only when interacting with some other genotypes [7, 16], has not been demonstrated in *M. xanthus*.)

Focusing on aggregative development as a cooperative process, the core phenotype in this system is how many spores strains produce across multiple social contexts, i.e. in clonal and mixed populations. Strains that produce proportionally more spores in a mixed population than they do in clonal culture show exploitation. Strains that produce significantly fewer spores in clonal populations than a wild-type or ancestral baseline are considered behaviorally obligate defectors. Defectors that have a positive relative fitness in a mixed population due to exploitation show cheating. By these criteria, exploitation is necessary but not sufficient to generate a cheating phenotype, because (i) non-defectors might exhibit exploitation and (ii) exploitation might or might not generate a relative-fitness advantage. These definitions of exploitation and cheating during social interactions do not require that the absolute fitness of exploited cooperators is reduced by such interactions. However, such reductions will often be expected, e.g. when obligate defectors present at a relatively high frequency in a population cheat on cooperators and thereby reduce total group productivity [2, 3, 7].

Fulfillment of the quantitative criteria for these definitions does not imply adaptation for increased proficiency at the observed behavior, e.g. adaptedness for exploitation or cheating. The questions of whether, in what manner, and to what degree an observed exploitative behavior evolved adaptively are distinct from the question of whether the behavior is observed in any particular context in the first place; such evolutionary questions require separate criteria and investigation.

### Bacterial strains and culturing

In the following assays, we examined pairwise social interactions among several lab strains and, separately, among several natural isolates of *M. xanthus*. These strains provide clear examples of (i) cheating by obligate defection, and (ii) exploitation between cooperators, respectively, in *M. xanthus*.

Lab strains. As the sporulation-proficient cooperator, we used *M. xanthus* strain GJV1 ([65]; strain S in [66], a derivative of DK1622 that is hereafter referred to as WT for “wild-type”). As obligately defecting cheaters, we used two strains derived from the MyxoEE-1 evolution experiment [67] (see also myxoee.org/myxoee1): GJV9 (a rifampicin-resistant variant of GVB207.3 [22]) and GVB206.3 (also rifampicin resistant [3]). Here, we refer to these cheaters as Ch1 and Ch2, respectively. Each was isolated from independently evolved populations descended from GJV1 after 1000 generations of growth in nutrient-rich liquid medium. Both have been repeatedly shown to cheat, that is to produce few or zero spores in pure culture while producing proportionally more spores than a cooperative partner in mixture [3, 7, 68, 69].

Natural isolates*.* For interactions between less-domesticated *M. xanthus* natural isolates, we used previously described antibiotic-resistant mutants of isolates DK801 (kanamycin-resistant) and Mxx41 (novobiocin-resistant) and the isolate Mxx144 (sensitive to both antibiotics), all of which sporulate at similar levels in monoculture [1]. In the study that first documented social exploitation during sporulation among these strains [1], DK801 and its mutant were collectively referred to as strain D, Mxx41 and its mutant were collectively referred to as strain G, and Mxx144 was referred to as strain I. For consistency with [1], hereafter in this study, we still refer to the resistant mutants of DK801 and Mxx41 as strains D and G, respectively, and Mxx144 as strain I. DK801 was isolated from California, United States; Mxx41 from Madras, India; Mxx144 from Mt. Ar-Li, Taiwan [1].

Growth conditions. In the following assays, we grew strains in either high-nutrient or low-nutrient conditions before inducing development. The high-nutrient condition (H) was CTT liquid [70], which contains 1% Casitone. The low-nutrient media (L) had the same composition as CTT except with reduced Casitone (0.1% for lab strains and 0.2% for natural isolates; some natural isolates did not grow sufficiently at the lower Casitone concentration). We grew the bacteria in liquid at 32°C and either 300 rpm (lab strains) or 200 rpm (natural isolates) and maintained them in exponential phase until starting the experiment.

### Developmental assays

**Figure 1:**
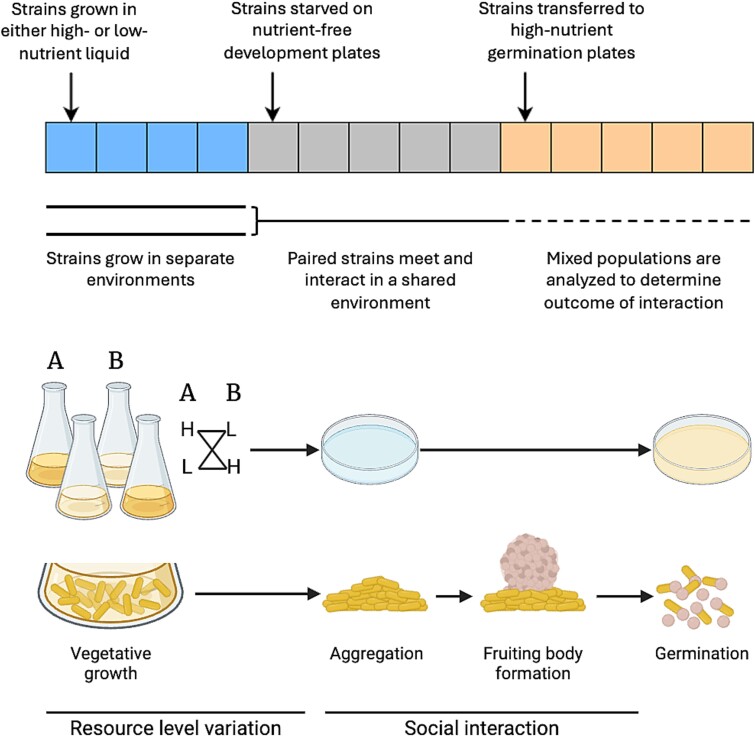
**Resource history and experimental design.** Here we show a timeline of the resource abundance experienced by the bacterial populations throughout the experiment, the methodological steps, and the respective life-cycle stages of the *Myxococcus xanthus* populations. Each segment of the timeline represents one day, and the color of the square represents the resource abundance blue (first four squares) = variable depending on treatment, grey (next five squares) = absent, orange (last five squares) = high). The flasks show strains A and B being grown under either high- or low-resource conditions and then being mixed pairwise under all resource-history combinations before plating onto nutrient-free plates. Our study asks whether the environmental conditions experienced by the strains when they grow separately (blue section of the timeline) influence the outcome of the subsequent interaction. Created with BioRender.com.

We grew all strains in high- and low-nutrient liquid media for 4 days (Fig. 1). We then centrifuged them at 5000x*g* for 15 minutes at 20°C and resuspended the pellets in TPM buffer [71] to a density of ~5 **×** 10^9^ cells/ml. For pure-culture assays, we spotted 100 μl of each culture onto TPM 1.5% agar plates. For lab-strain mixed-culture assays, we combined the cheater strain with the WT at a 1:9 ratio; for natural isolate mixes, we used a 1:1 ratio as in [1]. The 1:9 ratio roughly models a de novo mutant invading a cooperator population, and the 1:1 ratio roughly models established cooperator populations encountering each other in soil on equal terms. For each strain pair, we made mixes for every combination of resource history: H/H, H/L, L/H, and L/L. We spotted the entire 100 μl of each mixture onto TPM 1.5% agar plates. We incubated all plates at 32°C and 90% rH for 5 days. We then harvested each population into ddH_2_O, heating for 2 hours at 50°C to kill non-spore cells, and sonicating to disperse spores. We plated various dilutions of the spores into CTT 0.5% agar and incubated at 32°C and 90% rH until colonies were visible (5 days).

For lab strains, we plated spore samples in agar with and without 5 μg/ml rifampicin to obtain counts of cheater spores and total spores, respectively. For natural isolate mixes, we counted colonies of strain D in agar with 40 μg/ml kanamycin and colonies of strain G in agar with 10 μg/ml novobiocin; we estimated spore production by strain I in mixes by subtraction of selective-agar colony counts from non-selective-agar colony counts. When we counted no spores at the lower limit of detection, we estimated 0.9 spores. We performed four temporally separate replicates for all assays.

Using pure-culture sporulation assays, we tested whether the use of antibiotic as a selection agent alters the number of spores produced by D or G that germinate and grow into visible colonies (difference of means +/− antibiotic [95% confidence intervals], n = 4 for all; D_high_ = 0.31 [−0.37, 0.99], D_low_ = 0.18 [−0.65, 1.02], G_high_ = 0.01 [−0.20, 0.23], G_low_ = 0.07 [−0.27, 0.41]; we considered this reasonable evidence for equivalence and proceeded.

### Measures of cheating and exploitation

Cheating by lab strains. We tested for the ability of each known cheater strain to cheat on WT during sporulation for all four combinations of pre-development nutritional histories. We assessed cheating by calculating the relative fitness parameter *W_ij_* [63, 72]:


$$ {W}_{ij}={\mathit{\log}}_{10}\left(\frac{spores\ of\ strain\ i}{initial\ cells\ of\ strain\ i}\right)-{\mathit{\log}}_{10}\left(\frac{spores\ of\ strain\ j}{initial\ cells\ of\ strain\ j}\right). $$

Positive and negative values of this parameter indicate that a defector (strain *i*) exhibits higher or lower relative spore production than WT (strain *j*) in pairwise mixes, respectively. We say that cheating occurs in instances where a defector’s relative fitness is positive. Because the cheaters examined here make zero or extremely few spores by themselves, they can only achieve a positive *W_ij_* value over a sporulation-proficient strain by exploiting the latter, sporulating much more in mixture with the latter than they can alone.

Mixing effects among natural isolates. Following [1], we estimated for each strain the mixing-effect parameter *C_i_(j)* which compares the sporulation efficiency of a focal strain *i* in a pairwise-mixed competition with strain *j* relative to the sporulation efficiency of strain *i* in pure culture. The sporulation efficiency *D_i_* of strain *i* in pure culture is the ratio of cells that survive as viable spores: 


$$ {D}_i=\frac{N_i\left({t}_5\right)}{N_i\left({t}_0\right)} $$

*N_i_*(*t_5_*) is the viable population size after 5 days of development and *N_i_*(*t_0_*) is the viable population size before development, here 5 × 10^8^ cells.

The sporulation efficiency of strain *i* when mixed with strain *j* is given by


$$ {D}_i(j)=\frac{Ni(j)\left({t}_5\right)}{Ni(j)\left({t}_0\right)} $$

where *N_i_*(*t_0_*) is 2.5 × 10^8^ cells.

The effect of strain *j* on the sporulation of strain *i* when the two strains are mixed relative to sporulation by strain *i* in pure culture is given by


$$ {C}_i(j)=\mathit{\log}\left({D}_i(j)\right)-\mathit{\log}\left({D}_i\right) $$

Positive and negative values of C*_i_*(*j*) indicate that strain *i* generates proportionally more or fewer spores when mixed with strain *j* than when alone, respectively. Thus, in the context of *M. xanthus* sporulation, social exploitation by strain *i* in mixture with *j* is manifested as a positive C*_i_*(*j*) value. Mixing-effect values were calculated to test whether growth conditions affected mixing outcomes.

### Data analysis

We analyzed our data in R version 4.3.0 [73] and RStudio version 2023.03.0 [74] using the packages “tidyr” [75], “dplyr” [76], and “DescTools” [77], and we visualized them using the “ggplot2” package [78]. We first used generalized linear models to test for strain × nutrient-history effects on the response variable (log_10_(CFUs) for analysis of pure culture sporulation data, cheater *W_ij_* values for analysis of cheater:cooperator mixes, and *C_i_(j)* values of each partner for analysis of natural isolate mixes). We then asked two questions of each data set of strain mixes, using Bonferroni-Holm corrected *t*-tests, Tukey HSD tests, or Dunnett tests:

a) Did exploitation occur (is the value different from zero indicating a benefit or detriment of the interaction on the focal strain)?
b) Does nutrient history affect the interaction (comparing the values between nutrient-history treatments, including relative to H/H as a reference treatment)?

The latter directly addresses the effect of nutrient history on the interactions between the strains, and combining the two types of results allows us to identify cases where the effect of the environment altered the exploitation outcome. Original data and analysis scripts can be found at https://doi.org/10.5281/zenodo.10469400**.** For clarity, we generally present full statistical results in the supplement (Tables S1–S4) and reference only *p*-values in-line.

## Results

### Low-nutrient resource histories can prevent cheating

**Figure 2:**
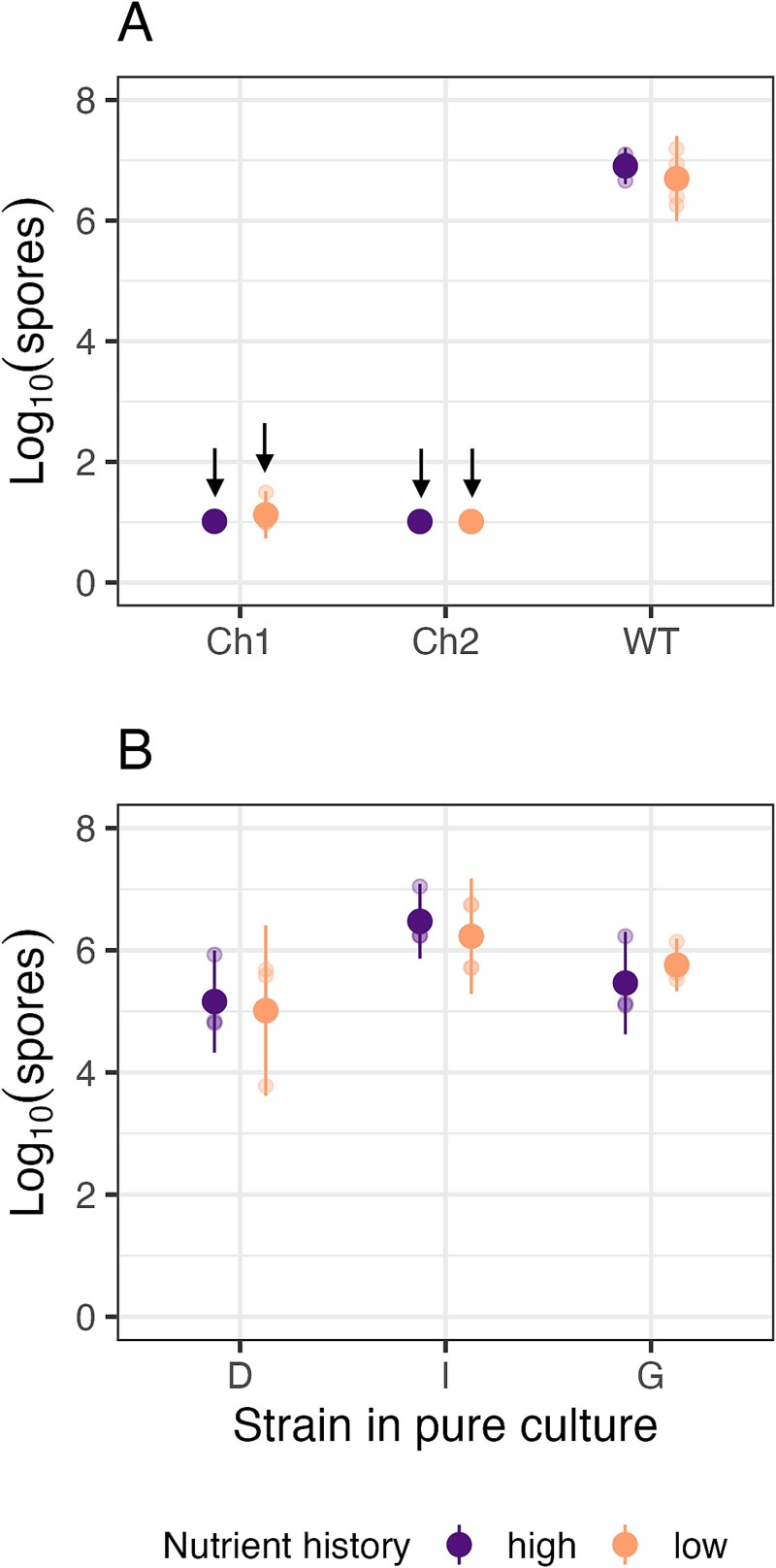
**Resource-level history does not alter spore production of single-genotype populations.** Total spore production in pure culture by two cheaters and a cooperator reference strain (A) and three natural isolates (B) used in this study after prior growth at high- or low-nutrient conditions. Small dots show individual-replicate estimates and large dots show cross-replicate means; 95% confidence intervals are represented. No strain showed a significant difference in sporulation ability between high vs low nutrient-level histories (Table S2, Test 1). Downward arrows indicate treatments in which zero spores were detected at the lower limit of detection (see *Methods*) in at least one replicate.

To understand effects of prior resource levels on outcomes of cheater-cooperator interactions during starvation-induced spore production, we first checked for effects in pure cultures of the cooperator WT and the two cheaters to establish the baseline effect of nutritional history. For all three strains, no effect of nutritional history was detected (Fig. 2A; Table S1 Test 1, *p* > .4), indicating that *M. xanthus’* ability to sporulate during starvation is independent of resource levels experienced prior to starvation. Any effect of resource history on outcomes in chimeric groups therefore depends on the interaction between strains.

We tested for effects of nutritional history on cheater vs. WT fitness outcomes in chimeric groups. Each cheater was mixed with WT in a 1:9 ratio in all four possible resource-history combinations (H/H, H/L, L/H, L/L). For each strain-pair and resource-history combination, we calculated the sporulation fitness of the respective cheater genotype relative to that of WT in the same mix, as reflected by the parameter *W_ij_* (see *Methods*). In these calculations, genotype *i* represents the cheater and genotype *j* represents WT. An ANOVA testing the effect of nutrient histories on cheater relative fitness (*W_ij_*) shows a significant interaction between cheater genotype and cheater nutrient history (Table 1, *p* = .04). Subsequent individual ANOVAs revealed significant effects of WT nutrient history on *W*_Ch1:WT_ and of both WT and Ch2 nutrient histories on *W*_Ch2:WT_ (Table S1 Tests 2–3, *p* ≤ .002). Overall, when one or both strains have a history of low resources, cheater fitness is reduced.

**Table 1:** ANOVA for effect of nutrient history on social interaction between cheaters and WT.

Test	Variable(s)	*F* statistic	*p* value
ANOVA for effect of cheater genotype × nutrients [Ch] × nutrients [WT] on *W_ij_*	cheater	F1,22 = 5.58	**.028**
	nutrients [Ch]	F1,22 = 25.28	**< .0001**
	nutrients [WT]	F1,22 = 34.05	**< .0001**
	cheater × nutrients [Ch]	F1,22 = 4.57	**.04**
	cheater × nutrients [WT]	F1,22 = 3.74	.06
	nutrients [Ch] × nutrients [WT]	F1,22 = 0.02	.88
	cheater × nutrients [Ch] × nutrients [WT]	F1,22 = 0.18	.67

**Figure 3:**
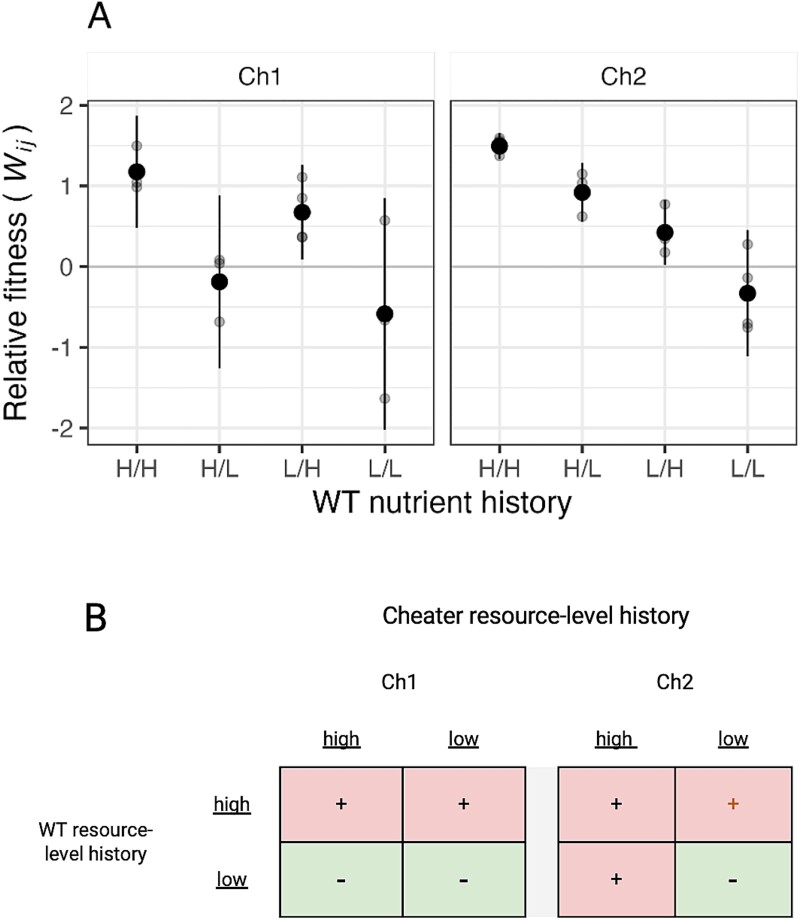
**Historical resource scarcity eliminates cheating in some strain-history combinations.** (A) Relative sporulation outcomes in pairwise mixes of each cheater strain with WT (*W_ij_*, *i =* cheater*; j =* WT*)* for all four possible resource-level-history combinations for each strain pair. H = high-nutrient history, L = low-nutrient history; the first letter shows the nutrient history of the cheater. Small dots show individual-replicate estimates and large dots show cross-replicate means; error bars show 95% confidence intervals. (B) Qualitative cheating-occurrence outcomes; whether cheating occurred (+) or not (−) is shown for all strain-pair/growth-history combinations. The brown + symbol in the upper right for Ch2 indicates a case in which cheating still occurred but cheater fitness was significantly lower than when both strains had grown at high resource levels.

We tested whether particular nutrient-history combinations determined whether cheating occurred or not. We say that cheating occurred in cases where *W_Ch:WT_* is significantly greater than zero (see *Methods*), i.e. the cheater produces proportionally more spores than WT in co-culture. In accordance with previous studies, the two cheaters exhibited strong cheating when both the cheaters and WT had grown under standard lab conditions (H/H), as reflected by significantly positive values of *W_Ch1-H:WT-H_* and *W_Ch2-H:WT-H_* (Fig. 3A; Table S2 Tests 4 & 5, *p* < .01).

We found that Ch1 was able to cheat whenever WT had grown under high-resource conditions, irrespective of Ch1’s own growth history (Fig. 3A; Table S2 Tests 4 & 6, *p* ≤ .04). However, Ch1 failed to cheat whenever WT had experienced low-nutrient conditions, again irrespective of Ch1 history (Fig. 3A; Table S2 Test 6, *p* = .86). For Ch2, in addition to cheating under H/H conditions (Fig. 3A; Table S2 Test 5, *p* < .001), cheating occurred if only Ch2 or WT had experienced low-nutrient conditions (*p* ≤ .04), but was not observed when both had experienced low-nutrient conditions (*p* = .86).

A history of low-nutrient growth for WT eliminated cheating by Ch1 regardless of Ch1’s nutritional history. Low-nutrient growth by WT also eliminated cheating by Ch2, but only when Ch2 had also experienced low-nutrient growth (Fig. 3B). Thus, the ecological history of a cooperator determined whether it was susceptible to cheating by two distinct cheaters, although exactly when WT history determined the occurrence of cheating (with respect to cheater history) differed across the two cheater genotypes.

### Low-nutrient history can reduce cheater performance even when cheating still occurs

We further analyzed the effects of resource history on cheater performance by testing for differences between *W_ij_* under H/H conditions versus under the non-standard conditions. Growing only the cheater with low nutrients had no effect on Ch1 fitness compared to H/H histories (Fig. 3A; Table S3 Test 7, *p* = .68), but it reduced relative fitness for Ch2 (Table S3 Test 8, *p* = .002) without eliminating cheating (Table S2 Test 7, *p* = .044).

In general, cheater relative fitness was reduced for both cheaters when WT was grown under low-nutrient conditions compared to WT growth at high resource levels (Figs. 3A, S1; Table S1 Tests 2 & 3, *p* ≤ .02). However, when only WT was grown with low nutrients (H/L), Ch2 performance may have been reduced relative to the Ch2-H/WT-H treatment, but the result was not significant (Table S3 Test 8, *p* = .8), and Ch2 still cheated (Table S2 Test 6, *p* = .012). We found similar results for Ch1 (Table S3 Test 7, *p* = .07), although in this case we did not find evidence of cheating (Table S2 Test 6, *p* = .86).

When both strains were grown with low nutrients, cheater performance was significantly reduced (Table S3 Tests 7 & 8, *p* ≤ .01). For Ch1, we don’t see a significant difference between Ch1-L/WT-L and either Ch1-H/WT-L (Table S3 Test 7, *p* = .81) or Ch1-L/WT-H (Table S3 Test 7, *p* = .05). However, for Ch2, there is a significant difference between Ch2-L/WT-L and both Ch2-H/WT-L (Table S3 Test 8, *p* = .0004) and Ch2-L/WT-H (Table S3 Test 8, *p* = .02).

To summarize, low-nutrient growth by one or both partners reduced cheater performance in some cases. For Ch1, this result is clear for the L/L condition, and there is the suggestion of an effect for Ch1-H/WT-L. There is evidence that Ch2 performance is reduced when either or both of the interacting strains has a prior history of lower nutrients, although the result for Ch2-H/WT-L is not quite significant; for Ch2-L/WT-H cheater performance is significantly reduced but cheating still occurs.

### Low-nutrient growth can prevent subsequent exploitation between natural isolates

To test for effects of nutrient availability during growth on exploitative relationships among three natural isolates during development, we compared the outcomes of pairwise competitions between the sporulation-proficient isolates for all four possible growth-history combinations (H/H, H/L, L/H, L/L). We calculated the mixing-effect parameter *C_i_*(*j*) (see *Methods*), where a positive value indicates exploitation of strain *j* by *i*. Irrespective of their nutrient-level history, all three strains sporulate at high levels in pure culture (Fig. 2), but strain I produces more spores than strains D and G (Fig. 2B; Table S4 Tests 9 & 10, *p* ≤ .03). As above, we tested for potential effects of nutritional history on subsequent developmental spore production in pure cultures. For all strains, no effect of nutrient level during growth was detected (Fig. 2B; Table S4 Test 11, *p* ≥ .6).

**Figure 4:**
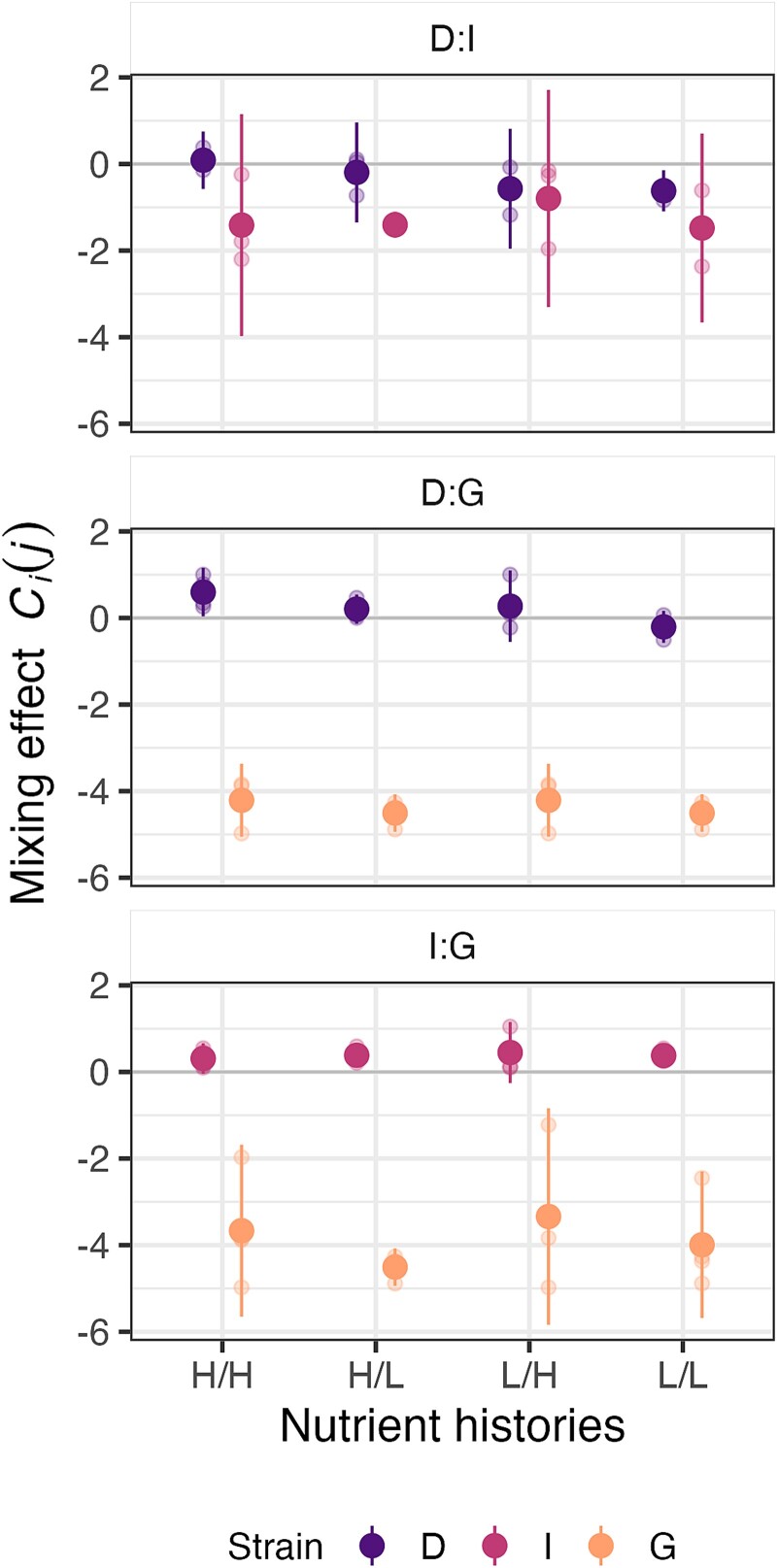
**Historical resource scarcity can reduce or eliminate exploitation between distinct cooperators.** The effects of pairwise mixing of two cooperation-proficient natural isolates on the sporulation efficiency of each paired strain—*C_i_(j)* (see *Methods*) —are shown for all four possible growth-history combinations for each of three strain pairs: (A) strains D vs G; (B) strains D vs I; (C) strains I vs G. *X*-axis labels identify the growth history of each partner (H = high-nutrient history, L = low-nutrient history). The first letter shows the nutrient history of the strain listed first above the panel. The first-listed strain of each pair is the strain previously shown to exploit the other after growth under standard lab conditions (H/H). The absence of any mixing effect on a given strain would correspond to a *C_i_(j)* value of 0; exploitative and antagonized responses to mixing are indicated by significantly positive and negative *C_i_(j)* values, respectively. Small dots show individual-replicate estimates and large dots show cross-replicate means; error bars represent 95% confidence intervals.

After growth under standard lab conditions (H/H), patterns of both relative spore production and social exploitation were generally similar to the outcomes of developmental competitions with these same strains conducted after H/H growth in a previous study [1], which demonstrated a clear fitness hierarchy (D > I > G). We again find clear evidence of strain G’s low relative fitness (Figs. 4, S2). Although strain D appears to have produced more spores than strain I in the H/H D:I mix, high variability in strain I spore estimates across replicates makes this unclear. Contrary to previous observation [1], apparent exploitation of strain I by strain D after H/H growth was not significant in our experiments (Fig. 4; Table S4 Test 12, *p* = .31). However, consistent with previous observations [1], strains D and I both exploited strain G (Fig. 4; Table S4 Tests 13 & 14, *p* ≤ .03).

Focusing first on the competitions between D and I, we found no evidence of D exploiting I in any of the three treatments in which at least one strain had been grown at the low resource level; all three *C_D_*(*I*) estimates were negative (Fig. 4). There was even a suggestion that D might be harmed in mixture with I under L/L conditions, although the result is not significant (Table S4 Test 15, *p* = .09). However, strain I was significantly harmed by interacting with D after D-H/I-L growth histories (Table S4 Test 15, *p* = .01). We found no significant evidence for changes in *C_i_*(*j*) due to any specific nutritional-history combination (Table S4 Tests 16 & 17, *p* ≥ 0.14).

In competitions between D and G, spore production by D (the strong winner, Fig. 4) was sensitive to the nutritional status of both strains. Although D exploited G under H/H conditions (as reported above, Fig. 4), strain D failed to show clear evidence of exploitation if one strain, but not the other, was grown under low resources (Table S4 Test 18, *p* ≥ 0.21). When both strains had previously experienced low nutrient levels, D was clearly unable to exploit G (Table S4 Test 19, *p* = .018). Spore production by G was extremely low in all mixtures, being reduced by a factor > 10^4^ (and to less than 0.01% of total spores; Fig. S2) under all conditions.

In mixes of strains I and G, mean *C_I_*(*G*) estimates suggest that strain I might exploit strain G in all growth-history treatments. *C_I_*(*G*) was significantly positive in three of the four treatments (Fig. 4; Table S4 Tests 13, 14 & 21, *p* ≤ .03) – including both treatments in which strain G had grown in low-nutrient medium – but not when strain I had grown under low nutrients and strain G had grown under high nutrients (Table S4 Test 21, *p*_I-L/G-H_ = 0.14). As in mixtures with strain D, G sporulated poorly in all treatments, consistently accounting for less than 0.1% of the total spore count (Fig. S2).

Although details differed, in several of the natural-isolate mixes, low-nutrient growth by either or both competitors reduced (or appeared to reduce) spore production by the dominant competitor relative to the respective H/H pairings. Thus, for some genotype combinations, low-nutrient growth before development in chimeric groups is found to prevent not only cheating by sporulation-defective strains (Fig. 3), but also social exploitation by sporulation-proficient natural isolates (Fig. 4). In our experiments, nutrient history often affected strain interactions without affecting the performance of individual strains in isolation (Fig. 2).

## Discussion

### Resource history determines whether cheating or exploitation happens

In pairs of a cooperation-proficient lab reference strain of *M. xanthus* and multiple cheater strains, and in pairs of natural isolates with only short histories of lab cultivation, the pre-development resource-level history of co-developing strains is found to strongly affect social fitness during sporulation. An abiotic ecological gradient changes the character of exploitative social interactions in microorganisms, just as abiotic gradients can alter interspecific interactions [49, 79, 80]. For three of five strain pairs examined, low-resource growth by one or both strains in the pair eliminated a clear exploitative interaction observed when both strains were grown with high resources. In one pairing with a cheater, low-nutrient growth by either party sufficed to prevent cheating, whereas in the other pair, cheating was only eliminated when both strains grew with low nutrients (Fig. 3). In one pair of high-sporulating natural isolates (D and G), low-nutrient growth by either competitor was sufficient to remove clear evidence of exploitation (Fig. 4).

### Mechanism hypotheses

Resource level during growth altered developmental competition outcomes by differentially impacting strain interactions rather than the intrinsic sporulation abilities of paired strains (Fig. 2). Several non-mutually exclusive hypotheses might explain why low-resource growth decreased the sporulation efficiency of cheaters relative to cooperators. These include differential effects of growth history on (a) developmental timing, (b) signal response, and/or (c) cell lysis. Regarding timing, low-resource history might increase the time it takes cheater cells to differentiate into spores compared to cooperator cells (without impacting final total productivity), thereby allowing the cooperator to convert more of its cells into spores in mixed groups than it can when the cheater has a history of high resources. The effect of low-nutrient conditions on sporulation would then be more pronounced in the early stages of development. This hypothesis could be tested by tracking temporal dynamics of spore production after growth in different media [56].

Regarding signaling, cheater cells in mixed groups with low–nutrient growth histories might be comparatively less responsive to developmental signals produced by the cooperator than they are in high-resource-history groups. This could manifest either in differences in sporulation timing (as outlined above) or in the conversion frequency of cheater cells into spores, irrespective of timing. Alternately, cooperators grown under low-resource conditions may not produce as many signal molecules as they do after high-resource growth, rendering them less cheatable. Regarding lysis, the comparative degree of cell lysis between high- vs low-resource-history in mixed groups might differ by genotype, thereby affecting relative fitness during interactions.

### Implications for natural populations

Our results complicate the effort to understand cheating, and social exploitation more broadly, in natural populations of microorganisms. They suggest that estimating the frequency of genotypes in a natural population that exhibit cheating under one set of ecological conditions might not accurately reflect the overall prevalence of cheating in that population across space and over time.

*M. xanthus* is a predator of fungi and other bacteria (reviewed in [81]). Wild strains of *M. xanthus* certainly encounter diverse potential prey in their natural environments and likely often vary in their ability to kill and consume any given prey genotype [82]. Our results suggest that if some co-developing cells experienced a nutrient-poor prey environment just before aggregation, this may, in some cases, reduce or prevent cheating. Cheating may therefore be less common under some natural conditions than would be inferred from studies performed under standard laboratory conditions with nutrient-rich growth. We suggest testing outcomes of social conflicts during development after co-developing parties had grown on prey which differ in nutritional accessibility toward *M. xanthus*. Together, our results suggest that the occurrence and strength (or degree) of cheating in nature may be highly variable across different ecological contexts, including different prey environments.

### Resource-level effects on cooperation vary across social systems

Whatever the mechanisms at play, it is clear that previous findings that high resource levels reduce cheating due to reduced costs of cooperation [32] do not reflect a universal rule of cooperative systems. Our results add to the body of work demonstrating that how distinct cooperative systems respond to environmental variables can differ [27, 83], even directionally. Effects of resource level on cooperation need further investigation across diverse systems until a broad picture of the degree of variation in such effects emerges.

An earlier study with the bacterium *Pseudomonas aeruginosa* [32] showed that increasing nutrient concentration can reduce the fitness of cheaters relative to cooperators in shaken liquid culture. The observed positive relationship between cooperator fitness and nutrient level in that study contrasts with our finding that low-nutrient histories often reduced cheater performance relative to high-nutrient histories during *M. xanthus* development, and even prevented cheating in some cases. There are multiple differences between these two studies that might contribute to their contrasting outcomes. First, in the previously published experiments, growth fueled by manipulated resource levels occurred simultaneously with the expression of the focal cooperative behavior. In our experiments, the manipulated resource environments were experienced historically—before starvation, during which cooperation occurred. How resource-level variation relates to the costs and benefits of cooperation may differ as a function of the timing of such variation relative to when cooperation occurs. Second, and relevant to temporal aspects, the cooperative trait of interest in our study—sporulation in the context of multicellular fruiting body development—is likely more mechanistically complex than the forms of public goods cooperation, such as siderophore production, that have been the focus of prior relevant studies. More complex forms of cooperation may be subject to a greater range of resource-level effects on interaction outcomes than simpler forms.

### Ecological history shapes social interaction

Species’ ecological histories can impact the outcomes of their interaction [50], in magnitude or even sign. For example, thermal histories can modify host–parasite-interaction phenotypes, such as when the degree to which *Spiroplasma* bacterial symbionts protect *Drosophila melanogaster* larvae from wasp attack is determined by the temperature at which the mother flies had previously developed, and not by the temperature at the time of wasp-larvae interaction [49]. In predator–prey interactions between distinct species of bacteria, the very direction of predation can reverse as a function of the temperature at which one of the parties grew before the interaction [51]. Here, we see that prior resource histories of conspecifics interacting during a cooperative process can determine both (i) whether cheating by genotypes obligately defective at cooperation occurs, and (ii) whether social exploitation between cooperation-proficient competitors occurs. These findings encourage further investigation of how ecological histories shape social interactions between genetically distinct conspecifics across diverse species in which individuals may experience ecologically distinct environments before cooperating. One of the aims of ecology is to identify which aspects of context are important. In this paper, we argue that prior nutrient history is relevant for exploitative interactions between (i) cheaters and cooperators and (ii) genetically distinct cooperators, in ways likely to influence evolution.

## Supplementary Material

2024_Schaal_et_al_Ecological_histories_SI_12Dec_wrae255

## Data Availability

The datasets generated during and/or analyzed during the current study are available in the Zenodo repository, https://doi.org/10.5281/zenodo.10469400.

## References

[1] Fiegna F, Velicer GJ. Exploitative and hierarchical antagonism in a cooperative bacterium. *PLoS Biol* 2005;3:e370. 10.1371/journal.pbio.0030370 16248676 PMC1275521

[2] Velicer GJ, Vos M. Sociobiology of the Myxobacteria. *Ann Rev Microbiol* 2009;63:599–623. 10.1146/annurev.micro.091208.073158 19575567

[3] Velicer GJ, Kroos L, Lenski RE. Developmental cheating in the social bacterium *Myxococcus xanthus*. *Nature* 2000;404:598–601. 10.1038/35007066 10766241

[4] Strassmann JE, Zhu Y, Queller DC. Altruism and social cheating in the social amoeba *Dictyostelium discoideum*. *Nature* 2000;408:965–7. 10.1038/35050087 11140681

[5] Diggle SP, Griffin AS, Campbell GS et al. Cooperation and conflict in quorum-sensing bacterial populations. *Nature* 2007;450:411–4. 10.1038/nature06279 18004383

[6] Ghoul M, Griffin AS, West SA. Toward an evolutionary definition of cheating. *Evolution* 2014;68:318–31. 10.1111/evo.12266 24131102

[7] Fiegna F, Velicer GJ. Competitive fates of bacterial social parasites: persistence and self–induced extinction of *Myxococcus xanthus* cheaters. *Proc R Soc B* 2003;270:1527–34. 10.1098/rspb.2003.2387 PMC1691394 12965020

[8] Jahan I, Larsen T, Strassmann JE et al. Group maintenance in aggregative multicellularity. In: Herron M.D., Conlin P.L., Ratcliff W.C. (eds.), The Evolution of Multicellularity. Boca Raton: CRC Press, 2022, 111–34.

[9] Harrison F . Cooperative production of siderophores by *Pseudomonas aeruginosa*. *Front Biosci* 2009;Volume:4113–26. 10.2741/3516 19273338

[10] Doebeli M, Hauert C. Models of cooperation based on the Prisoner’s dilemma and the snowdrift game. *Ecol Lett* 2005;8:748–66. 10.1111/j.1461-0248.2005.00773.x

[11] Lehmann L, Keller L. The evolution of cooperation and altruism—a general framework and a classification of models. *J Evol Biol* 2006;19:1365–76. 10.1111/j.1420-9101.2006.01119.x 16910958

[12] Hamilton WD . The genetical evolution of social behaviour. *I J Theor Biol* 1964;7:1–16. 10.1016/0022-5193(64)90038-4 5875341

[13] Frank SA . Perspective: repression of competition and the evolution of cooperation. *Evolution* 2003;57:693–705. 10.1554/0014-3820(2003)057[0693:PROCAT]2.0.CO;2 12778541

[14] Van Dyken JD, Linksvayer TA, Wade MJ. Kin selection-mutation balance: a model for the origin, maintenance, and consequences of social cheating. *Am Nat* 2011;177:288–300. 10.1086/658365 21460538

[15] Nair RR, Fiegna F, Velicer GJ. Indirect evolution of social fitness inequalities and facultative social exploitation. *Proc R Soc B* 2018;285:20180054. 10.1098/rspb.2018.0054 PMC5897644 29593113

[16] Buttery NJ, Rozen DE, Wolf JB et al. Quantification of social behavior in *D. Discoideum* reveals complex fixed and facultative strategies. *Curr Biol* 2009;19:1373–7. 10.1016/j.cub.2009.06.058 19631539

[17] Branda SS, Gonzalez-Pastor JE, Ben-Yehuda S et al. Fruiting body formation by *Bacillus subtilis*. *Proc Natl Acad Sci* 2001;98:11621–6. 10.1073/pnas.191384198 11572999 PMC58779

[18] Crespi BJ . The evolution of social behavior in microorganisms. *Trends Ecol Evol* 2001;16:178–83. 10.1016/S0169-5347(01)02115-2 11245940

[19] Fiegna F, Pande S, Peitz H et al. Widespread density dependence of bacterial growth under acid stress. *iScience* 2023;26:106952. 10.1016/j.isci.2023.106952 37332671 PMC10275722

[20] Griffin AS, West S, Buckling A. Cooperation and competition in pathogenic bacteria. *Nature* 2004;430:1024–7. 10.1038/nature02744 15329720

[21] Harrison F, Buckling A. Siderophore production and biofilm formation as linked social traits. *ISME J* 2009;3:632–4. 10.1038/ismej.2009.9 19225554

[22] Manhes P, Velicer GJ. Experimental evolution of selfish policing in social bacteria. *Proc Natl Acad Sci* 2011;108:8357–62. 10.1073/pnas.1014695108 21531905 PMC3100924

[23] McGrath S, Wade DS, Pesci EC. Dueling quorum sensing systems in *Pseudomonas aeruginosa* control the production of the *pseudomonas* quinolone signal (PQS). *FEMS Microbiol Lett* 2004;230:27–34. 10.1016/S0378-1097(03)00849-8 14734162

[24] Pande S, Pérez Escriva P, Yu YTN et al. Cooperation and cheating among germinating spores. *Curr Biol* 2020;30:4745–4752.e4. 10.1016/j.cub.2020.08.091 32976811

[25] Pesci EC, Iglewski BH, Latifi A et al. The chain of command in *pseudomonas* quorum sensing. *Trends Microbiol* 1997;5:132–4. 10.1016/S0966-842X(97)01008-1 9141185

[26] Rainey P, Rainey K. Evolution of cooperation and conflict in experimental bacterial populations. *Nature* 2003;425:72–4. 10.1038/nature01906 12955142

[27] Ross-Gillespie A, Gardner A, Buckling A et al. Density dependence and cooperation: theory and a test with bacteria. *Evolution* 2009;63:2315–25. 10.1111/j.1558-5646.2009.00723.x 19453724

[28] Sandoz KM, Mitzimberg SM, Schuster M. Social cheating in *Pseudomonas aeruginosa* quorum sensing. *Proc Natl Acad Sci* 2007;104:15876–81. 10.1073/pnas.0705653104 17898171 PMC2000394

[29] Shapiro JA . Thinking about bacterial populations As multicellular organisms. *Ann Rev Microbiol* 1998;52:81–104. 10.1146/annurev.micro.52.1.81 9891794

[30] Wilder CN, Diggle SP, Schuster M. Cooperation and cheating in *Pseudomonas aeruginosa*: the roles of the *las*, *rhl* and *pqs* quorum-sensing systems. *ISME J* 2011;5:1332–43. 10.1038/ismej.2011.13 21368905 PMC3146268

[31] Zusman DR, Scott AE, Yang Z et al. Chemosensory pathways, motility and development in *Myxococcus xanthus*. *Nat Rev Microbiol* 2007;5:862–72. 10.1038/nrmicro1770 17922045

[32] Brockhurst MA, Buckling A, Racey DA et al. Resource supply and the evolution of public-goods cooperation in bacteria. *BMC Biol* 2008;6:20. 10.1186/1741-7007-6-20 18479522 PMC2409295

[33] Brockhurst MA, Colegrave N, Rozen DE. Next-generation sequencing as a tool to study microbial evolution. *Mol Ecol* 2011;20:972–80. 10.1111/j.1365-294X.2010.04835.x 20874764

[34] Harrison F, Paul J, Massey RC et al. Interspecific competition and siderophore-mediated cooperation in *Pseudomonas aeruginosa*. *ISME J* 2008;2:49–55. 10.1038/ismej.2007.96 18180746

[35] Kümmerli R, Jiricny N, Clarke LS et al. Phenotypic plasticity of a cooperative behaviour in bacteria. *J Evol Biol* 2009;22:589–98. 10.1111/j.1420-9101.2008.01666.x 19170825

[36] Vasse M, Noble RJ, Akhmetzhanov AR et al. Antibiotic stress selects against cooperation in the pathogenic bacterium *Pseudomonas aeruginosa*. *Proc Natl Acad Sci USA* 2017;114:546–51. 10.1073/pnas.1612522114 28049833 PMC5255613

[37] O’Brien S, Hodgson DJ, Buckling A. Social evolution of toxic metal bioremediation in *Pseudomonas aeruginosa*. *Proc R Soc B* 2014;281:20140858. 10.1098/rspb.2014.0858 PMC4071558 24898376

[38] García-Contreras R, Nuñez-López L, Jasso-Chávez R et al. Quorum sensing enhancement of the stress response promotes resistance to quorum quenching and prevents social cheating. *ISME J* 2015;9:115–25. 10.1038/ismej.2014.98 24936763 PMC4274435

[39] Kümmerli R, Griffin AS, West SA et al. Viscous medium promotes cooperation in the pathogenic bacterium *Pseudomonas aeruginosa*. *Proc R Soc B Biol Sci* 2009;276:3531–8. 10.1098/rspb.2009.0861 PMC2817189 19605393

[40] O’Brien S, Culbert C, Barraclough TG. Public goods exploitation is reduced in species-rich microbial communities. *bioRxiv* 2022. 10.1101/2022.11.25.517952

[41] Friman VP, Diggle SP, Buckling A. Protist predation can favour cooperation within bacterial species. *Biol Lett* 2013;9:20130548. 10.1098/rsbl.2013.0548 23945212 PMC3971697

[42] Morgan AD, Quigley BJZ, Brown SP et al. Selection on non-social traits limits the invasion of social cheats. *Ecol Lett* 2012;15:841–6. 10.1111/j.1461-0248.2012.01805.x 22639835 PMC3444687

[43] Saucedo-Mora MA, Castañeda-Tamez P, Cazares A et al. Selection of functional quorum sensing systems by lysogenic bacteriophages in *Pseudomonas aeruginosa*. *Front Microbiol* 2017;8:1669. 10.3389/fmicb.2017.01669 28912771 PMC5583629

[44] Vasse M, Torres-Barceló C, Hochberg ME. Phage selection for bacterial cheats leads to population decline. *Proc R Soc B* 2015;282:20152207. 10.1098/rspb.2015.2207 PMC4650167 26538598

[45] O’Brien S, Kümmerli R, Paterson S et al. Transposable temperate phages promote the evolution of divergent social strategies in *Pseudomonas aeruginosa* populations. *Proc R Soc B* 2019;286:20191794. 10.1098/rspb.2019.1794 PMC6790780 31594506

[46] Sexton DJ, Schuster M. Nutrient limitation determines the fitness of cheaters in bacterial siderophore cooperation. *Nat Commun* 2017;8:230. 10.1038/s41467-017-00222-2 28794499 PMC5550491

[47] Fronhofer EA, Liebig J, Mitesser O et al. Eusociality outcompetes egalitarian and solitary strategies when resources are limited and reproduction is costly. *Ecol Evol* 2018;8:12953–64. 10.1002/ece3.4737 30619596 PMC6309011

[48] McGhee KE, Pintor LM, Suhr EL et al. Maternal exposure to predation risk decreases offspring antipredator behaviour and survival in threespined stickleback. *Funct Ecol* 2012;26:932–40. 10.1111/j.1365-2435.2012.02008.x 22962510 PMC3434968

[49] Jones JE, Hurst GDD. History matters: thermal environment before but not during wasp attack determines the efficiency of symbiont-mediated protection. *Mol Ecol* 2023;32:3340–51. 10.1111/mec.16935 36946891

[50] Grigaltchik VS, Ward AJW, Seebacher F. Thermal acclimation of interactions: differential responses to temperature change alter predator-prey relationship. *Proc R Soc B Biol Sci* 2012;279:4058–64. 10.1098/rspb.2012.1277 PMC3427582 22859598

[51] Vasse M, Fiegna F, Kriesel B et al. Killer prey: ecology reverses bacterial predation. *PLoS Biol* 2024;22:e3002454. 10.1371/journal.pbio.3002454 38261596 PMC10805292

[52] Dubravcic D, van Baalen M, Nizak C. An evolutionarily significant unicellular strategy in response to starvation stress in *Dictyostelium* social amoebae. *F1000Research* 2014;3:133. 10.12688/f1000research.4218.1 25309731 PMC4184345

[53] Forget M, Adiba S, De Monte S. Social conflicts in *Dictyostelium discoideum* : a matter of scales. *Peer Community J* 2021;1:e58. 10.24072/pcjournal.39

[54] Kessin RH . Chapter 9: Differentiation and adhesion in the aggregate. Dictyostelium: Evolution, Cell Biology, and the Development of Multicellularity. Cambridge, UK: Cambridge University Press, 2001.

[55] Kessin RH . Chapter 10: The behavior of cells in the slug. In: Dictyostelium: Evolution, Cell Biology, and the Development of Multicellularity. Cambridge, UK: Cambridge University Press, 2001.

[56] Kuzdzal-Fick JJ, Queller DC, Strassmann JE. An invitation to die: initiators of sociality in a social amoeba become selfish spores. *Biol Lett* 2010;6:800–2. 10.1098/rsbl.2010.0257 20504816 PMC3001359

[57] Castillo DI, Queller DC, Strassmann JE. Cell condition, competition, and chimerism in the social amoeba *Dictyostelium discoideum*. *Ethol Ecol Evol* 2011;23:262–73. 10.1080/03949370.2011.568526

[58] Leach CK, Ashworth JM, Garrod DR. Cell sorting out during the differentiation of mixtures of metabolically distinct populations of *Dictyostelium discoideum*. *J Embryol Exp Morphol* 1973;29:647–61. 4736935

[59] Kroos L . Highly signal-responsive gene regulatory network governing *Myxococcus* development. *Trends Genet* 2017;33:3–15. 10.1016/j.tig.2016.10.006 27916428 PMC5182100

[60] Yang Z., Higgs P.I. (eds.). Myxobacteria: Genomics, Cellular and Molecular Biology. Poole, UK: Caister Academic Press, 2014.

[61] Lee B, Holkenbrink C, Treuner-Lange A et al. *Myxococcus xanthus* developmental cell fate production: heterogeneous accumulation of developmental regulatory proteins and reexamination of the role of MazF in developmental lysis. *J Bacteriol* 2012;194:3058–68. 10.1128/JB.06756-11 22493014 PMC3370845

[62] Amherd M, Velicer GJ, Rendueles O. Spontaneous nongenetic variation of group size creates cheater-free groups of social microbes. *Behav Ecol* 2018;29:393–403. 10.1093/beheco/arx184

[63] Schaal KA, Yu Y-TN, Vasse M et al. Allopatric divergence of cooperators confers cheating resistance and limits effects of a defector mutation. *BMC Ecol Evol* 2022;22:141. 10.1186/s12862-022-02094-7 36510120 PMC9746145

[64] Vos M, Velicer GJ. Social conflict in Centimeter-and global-scale populations of the bacterium *Myxococcus xanthus*. *Curr Biol* 2009;19:1763–7. 10.1016/j.cub.2009.08.061 19879146 PMC2858270

[65] Velicer GJ, Raddatz G, Keller H et al. Comprehensive mutation identification in an evolved bacterial cooperator and its cheating ancestor. *Proc Natl Acad Sci* 2006;103:8107–12. 10.1073/pnas.0510740103 16707573 PMC1472437

[66] Velicer GJ, Kroos L, Lenski RE. Loss of social behaviors by *Myxococcus xanthus* during evolution in an unstructured habitat. *Proc Natl Acad Sci* 1998;95:12376–80. 10.1073/pnas.95.21.12376 9770494 PMC22839

[67] Rendueles O, Velicer GJ. Hidden paths to endless forms most wonderful: complexity of bacterial motility shapes diversification of latent phenotypes. *BMC Evol Biol* 2020;20:145. 10.1186/s12862-020-01707-3 33148179 PMC7641858

[68] Velicer GJ, Stredwick KL. Experimental social evolution with *Myxococcus xanthus*. *Antonie Van Leeuwenhoek Int J Gen Mol Microbiol* 2002;81:155–64. 10.1023/A:1020546130033 12448714

[69] Fiegna F, Yu YN, Kadam SV et al. Evolution of an obligate social cheater to a superior cooperator. *Nature* 2006;441:310–4. 10.1038/nature04677 16710413

[70] Hodgkin J, Kaiser D. Cell-to-cell stimulation of movement in nonmotile mutants of *Myxococcus*. *Proc Natl Acad Sci* 1977;74:2938–42. 10.1073/pnas.74.7.2938 16592422 PMC431354

[71] Kuspa A, Kroos L, Kaiser D. Intercellular signaling is required for developmental gene expression in *Myxococcus xanthus*. *Dev Biol* 1986;117:267–76. 10.1016/0012-1606(86)90369-6 3017795

[72] Manhes P, Schaal KA, Velicer GJ. Antagonistic, synergistic, and social pleiotropy in microbial cheaters. *bioRxiv* 2022. 10.1101/2022.08.11.503674

[73] R Core Team . R: A Language and Environment for Statistical Computing. R Foundation for Statistical Computing. Vienna: Austria, 2018.

[74] RStudio Team. RStudio: Integrated Development for R . Studio. Boston, MA: PBC, 2020.

[75] Wickham H, Vaughan D, Girlich M. Tidyr: Tidy Messy Data. R package version 1.3.1. 2024. https://github.com/tidyverse/tidyr, https://tidyr.tidyverse.org

[76] Wickham H, François R, Henry L et al. Dplyr: A Grammar of Data Manipulation. R package version 1.1.4. 2023. https://github.com/tidyverse/dplyr, https://dplyr.tidyverse.org

[77] Signorell A et al. DescTools: Tools for descriptive statistics. R package version 0.99.23. 2017.

[78] Wickham H. ggplot2: Elegant Graphics for Data Analysis. Springer-Verlag New York, 2016. 10.1007/978-3-319-24277-4.

[79] Johnson NC, Graham JH, Smith FA. Functioning of mycorrhizal associations along the mutualism-parasitism continuum. *New Phytol* 1997;135:575–85. 10.1046/j.1469-8137.1997.00729.x

[80] Friede M, Unger S, Hellmann C et al. Conditions promoting mycorrhizal parasitism are of minor importance for competitive interactions in two differentially mycotrophic species. *Front Plant Sci* 2016;7:1465. 10.3389/fpls.2016.01465 27729924 PMC5037182

[81] Thiery S, Kaimer C. The predation strategy of *Myxococcus xanthus*. *Front Microbiol* 2020;11:2. 10.3389/fmicb.2020.00002 32010119 PMC6971385

[82] Morgan AD, MacLean RC, Hillesland KL et al. Comparative analysis of *Myxococcus* predation on soil bacteria. *Appl Environ Microbiol* 2010;76:6920–7. 10.1128/AEM.00414-10 20802074 PMC2953020

[83] Van Dyken JD, Wade MJ. Origins of altruism diversity I: the diverse ecological roles of altruistic strategies and their evolutionary responses to local competition. *Evolution* 2012;66:2484–97. 10.1111/j.1558-5646.2012.01630.x 22834747 PMC3408632

