# Supplementary material for: Ecological histories govern social exploitation by microorganisms
Source: ISME J. 2024 Dec 23;19(1):wrae255. doi: 10.1093/ismejo/wrae255 (PMC11831026; doi:10.1093/ismejo/wrae255)
Supplement: 2024_Schaal_et_al_Ecological_histories_SI_12Dec_wrae255 [file 2024_schaal_et_al_ecological_histories_si_12dec_wrae255.docx]

**Supplemental Information for:**

**Ecological histories govern social exploitation by microorganisms**

Contents:

Figure S1: Absolute spore production by cheaters and WT in mixture

Figure S2: Spore production by natural isolates in mixture

Tables S1-S4: Statistical tests and results


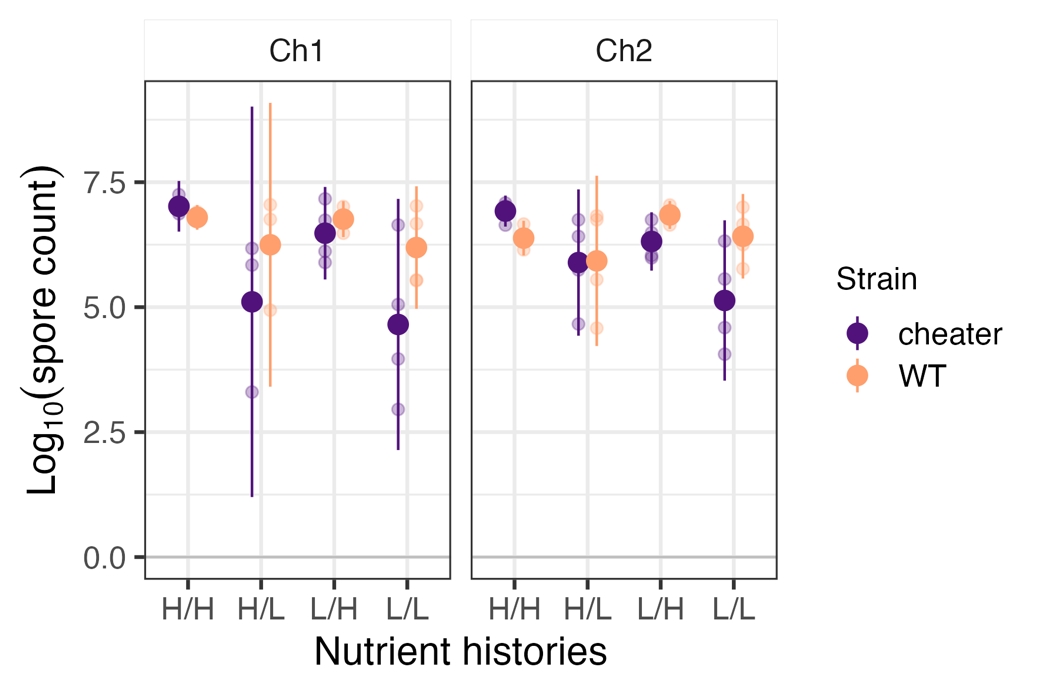


**Figure S1.** **Absolute spore production by cheaters and WT in mixture**. Spore production values by cheaters in mixture with WT used to calculate *W_ij_* values in Figure 3. H = high-nutrient history, L = low-nutrient history; the first letter shows the nutrient history of the strain listed first above the panel. Small dots show individual-replicate estimates and large dots show cross-replicate means; error bars represent 95% confidence intervals.


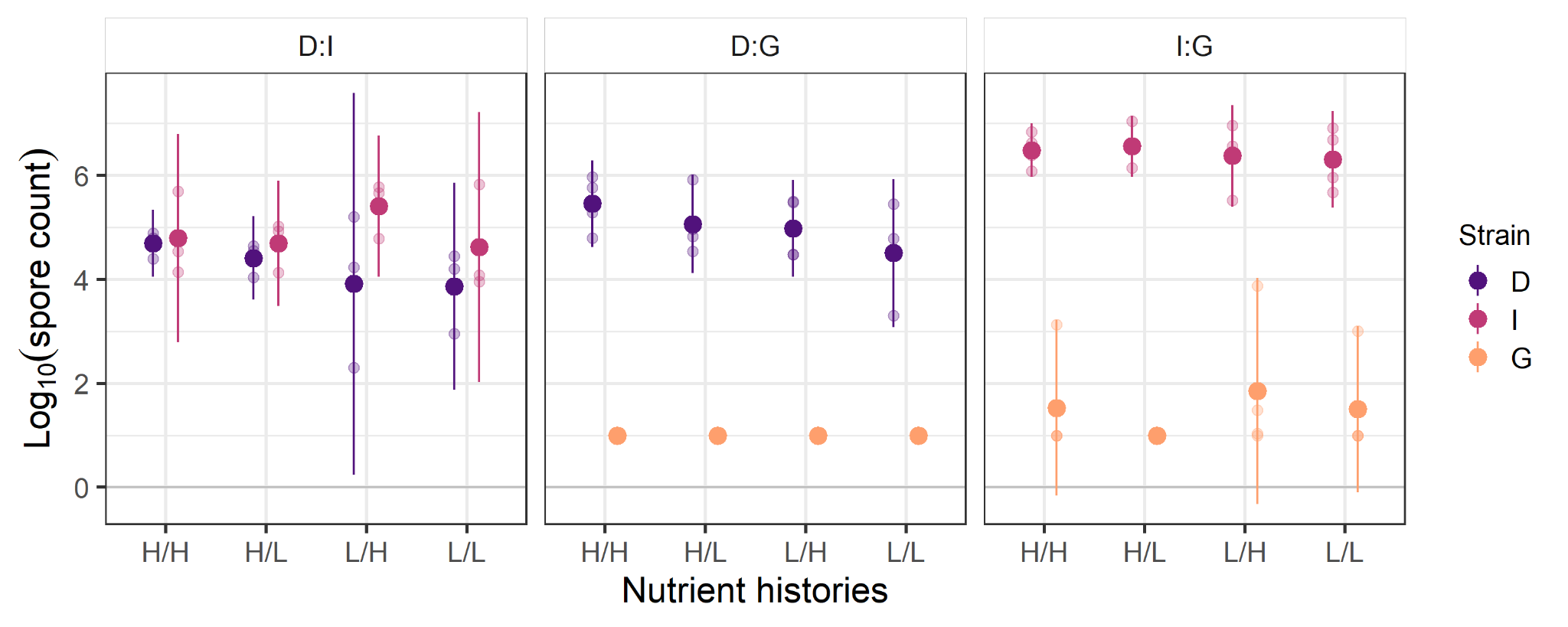


**Figure S2. Spore production by natural isolates in mixture**. Spore production values by natural isolates in pairwise mixes used to calculate *C_i_(j)* values for Figure 4. Spore counts for strain I were calculated by subtracting counts for either D or G from total counts obtained from nonselective dilution plates. H = high-nutrient history, L = low-nutrient history; the first letter shows the nutrient history of the strain listed first above the panel. Small dots show individual-replicate estimates and large dots show cross-replicate means; error bars represent 95% confidence intervals.

| **Table S1. Statistical tests for effects of nutrient histories (‘nutrients’) on pure-culture spore production (Test 1) and cheater *W_ij_* values in competition with WT (Tests 2 and 3)** | | | | |
| --- | --- | --- | --- | --- |
| # | Test | Variable(s) | F-statistic | p-value |
| 1 | ANOVA for effect of strain x nutrients on log_10_(CFUs) | strain | F_2,17_ = 1673.17 | < 0.0001 |
|  |  | nutrients | F_1,17_ = 0.18 | 0.68 |
|  |  | strain x nutrients | F_2,17_ = 0.95 | 0.41 |
| 2 | ANOVA for effect of Ch1 nutrients x WT nutrients on *W_ij_* of Ch1 | nutrients[Ch1] | F_1,10_ = 2.06 | 0.18 |
|  |  | nutrients[WT] | F_1,10_ = 17.56 | 0.002 |
|  |  | nutrients[Ch1] x nutrients[WT] | F_1,10_ = 0.03 | 0.87 |
| 3 | ANOVA for effect of Ch2 nutrients x WT nutrients on *W_ij_* of Ch2 | nutrients[Ch2] | F_1,12_ = 58.7 | < 0.0001 |
|  |  | nutrients[WT] | F_1,12_ = 19.2 | 0.0009 |
|  |  | nutrients[Ch2] x nutrients[WT] | F_1,12_ = 0.35 | 0.56 |

| **Table S2. Statistical tests for whether cheating occurred in each strain pair x nutrient history combination** | | | | |
| --- | --- | --- | --- | --- |
| # | Test | Notes | t-statistic & df | p-value |
| 4 | One Sample t-test of whether *W_Ch1:WT_* > 0, H/H histories | Does cheating happen? A priori = yes | t = 7.28, df = 2 | 0.009 |
| 5 | One Sample t-test of whether *W_Ch2:WT_* > 0, H/H histories | Does cheating happen? A priori = yes | t = 29.75, df = 3 | < 0.0001 |
| 6 | One Sample t-tests of whether *W_Ch:WT_* > 0 for H/L, L/H and L/L histories; Bonferroni-Holm corrected | Ch1 H/L | t = -0.75, df = 2 | 0.86 |
|  |  | Ch1 L/H | t = 3.64, df = 3 | 0.04 |
|  |  | Ch1 L/L | t = -1.3, df = 3 | 0.86 |
|  |  | Ch2 H/L | t = 8.05, df = 3 | 0.01 |
|  |  | Ch2 L/H | t = 3.36, df = 3 | 0.04 |
|  |  | Ch2 L/L | t = -1.34, df = 3 | 0.86 |

| **Table S3. Statistical tests for differences in *W_ij_* values between distinct nutrient history combinations** | | | | |
| --- | --- | --- | --- | --- |
| # | Test | Comparison | difference in means  [95% confidence interval] | p-value |
| 7 | Post-hoc Tukey HSD tests from Test 2 (*W_ij_* of Ch1) | H-H - L/H | 0.5 [-0.86, 1.86] | 0.68 |
|  |  | H/H - H/L | 1.36 [-0.09, 2.82] | 0.07 |
|  |  | H/H - L/L | 1.76 [0.4, 3.12] | 0.01 |
|  |  | L/H - H/L | 0.86 [-0.5, 2.22] | 0.27 |
|  |  | L/H - L/L | 1.25 [-0.0006, 2.52] | 0.05 |
|  |  | H/L - L/L | 0.4 [-0.96, 1.76] | 0.81 |
| 8 | Post-hoc Tukey HSD tests from Test 3 (*W_ij_* of Ch2) | H-H - L/H | 1.07 [0.43, 1.71] | 0.002 |
|  |  | H/H - H/L | 0.57 [-0.06, 1.21] | 0.08 |
|  |  | H/H - L/L | 1.82 [1.19, 2.46 | < 0.0001 |
|  |  | L/H - H/L | 0.5 [-1.13, 0.13] | 0.15 |
|  |  | L/H - L/L | 0.75 [0.12, 1.39] | 0.02 |
|  |  | H/L - L/L | 1.25 [0.61, 1.89] | 0.0004 |

| **Table S4. Statistical tests for effect of resource history on exploitation in natural isolates** | | | | |
| --- | --- | --- | --- | --- |
| # | Test |  |  | p-value |
| 9 | ANOVA for differences in log_10_(CFUs) among isolates | strain | F_2,21_ = 11.27 | 0.0005 |
| 10 | Post-hoc Tukey HSD tests from Test 9 | I - D | 1.27 [0.59, 1.94] | 0.0003 |
|  |  | I - G | 0.74 [0.06, 1.42] | 0.03 |
|  |  | G - D | 0.53 [-0.15, 1.2] | 0.15 |
| 11 | ANOVA for effect of strain x nutrients on log_10_(CFUs) | nutrients | F_1,18_ = 0.021 | 0.89 |
|  |  | strain | F_2,18_ = 10.24 | 0.001 |
|  |  | nutrients x strain | F_2,18_ = 0.53 | 0.6 |
| 12 | One Sample t-test, µ > 0, *C_D_(I)* under H/H | Does D exploit I? A priori = yes | t = 0.06, df = 2 | 0.31 |
| 13 | One Sample t-test, µ > 0, *C_D_(G)* under H/H | Does D exploit G? A priori = yes | t = 3.39, df = 3 | 0.02 |
| 14 | One Sample t-test, µ > 0, *C_I_(G)* under H/H | Does I exploit G? A priori = yes | t = 2.81, df = 3 | 0.03 |
| 15 | One Sample t-tests, Bonferroni-Holm corrected, is *C_i_(j)* different from 0, D:I, H/L & L/H & L/L | **H**/L (D) | t = -0.72, df = 2 | 0.54 |
|  |  | H/**L** (I) | t = -20.22, df = 2 | 0.01 |
|  |  | **L**/H (D) | t = -1.77, df = 2 | 0.33 |
|  |  | L/**H** (I) | t = -1.37, df = 2 | 0.36 |
|  |  | **L**/L (D) | t = -5.6, df = 2 | 0.09 |
|  |  | L/**L** (I) | t = -2.9, df = 2 | 0.2 |
| 16 | Dunnett test for D mixed with I, control = H/H | H/L - H/H | -0.28 [-1.22, 0.66] | 0.73 |
|  |  | L/H - H/H | -0.66 [-1.6, 0.27] | 0.18 |
|  |  | L/L - H/H | -0.71 [1.64, 0.23] | 0.14 |
| 17 | Dunnett test for I mixed with D, control = H/H | H/L - H/H | 0.007 [-1.98, 2] | 1 |
|  |  | L/H - H/H | 0.61 [-1.38, 2.61] | 0.71 |
|  |  | L/L - H/H | -0.07 [-2.06, 1.92] | 1 |
| 18 | One Sample t-tests, Bonferroni-Holm corrected, is *C_i_(j)* different from 0, D:G, H/L & L/H & L/L | **H**/L (D) | t = 1.94, df = 3 | 0.21 |
|  |  | H/**L** (G) | t = -33.18, df = 3 | 0.0002 |
|  |  | **L**/H (D) | t = 1.05, df = 3 | 0.37 |
|  |  | L/**H** (G) | t = -15.95, df = 3 | 0.011 |
|  |  | **L**/L (D) | t = -1.77, df = 3 | 0.21 |
|  |  | L/**L** (G) | t = -33.18, df = 3 | 0.0002 |
| 19 | Dunnett test for D mixed with G, control = H/H | H/L - H/H | -0.39 [-1.06, 0.27] | 0.31 |
|  |  | L/H - H/H | -0.32 [-0.99, 0.34] | 0.45 |
|  |  | L/L - H/H | -0.80 [-1.47, -0.14] | 0.018 |
| 20 | Dunnett test for G mixed with D, control = H/H | H/L - H/H | -0.30 [-1.09, 0.5] | 0.64 |
|  |  | L/H - H/H | 0.00 [-0.80, 0.80] | 1 |
|  |  | L/L - H/H | -0.30 [-1.09, 0.5] | 0.64 |
| 21 | One Sample t-tests, Bonferroni-Holm corrected, is *C_i_(j)* different from 0, I:G, H/L & L/H & L/L | **H**/L (I) | t = 4.66, df = 3 | 0.028 |
|  |  | H/**L** (G) | t = -33.18, df = 3 | 0.0004 |
|  |  | **L**/H (I) | t = 2.02, df = 3 | 0.14 |
|  |  | L/**H** (G) | t = -4.25, df = 3 | 0.029 |
|  |  | **L**/L (I) | t = 5.25, df = 3 | 0.027 |
|  |  | L/**L** (G) | t = -7.51, df = 3 | 0.015 |
| 22 | Dunnett test for I mixed with G, control = H/H | H/L - H/H | 0.07 [-0.44, 0.59] | 0.96 |
|  |  | L/H - H/H | 0.13 [-0.38, 0.65] | 0.82 |
|  |  | L/L - H/H | 0.06 [-0.44, 0.58] | 0.97 |
| 23 | Dunnett test for G mixed with I, control = H/H | H/L - H/H | -0.84 [-3.01, 1.33] | 0.61 |
|  |  | L/H - H/H | 0.33 [-1.84, 2.50] | 0.96 |
|  |  | L/L - H/H | -0.32 [-2.49, 1.84] | 0.96 |
